# *In Vivo* Gene Knockdown in Rat Dorsal Root Ganglia Mediated by Self-Complementary Adeno-Associated Virus Serotype 5 Following Intrathecal Delivery

**DOI:** 10.1371/journal.pone.0032581

**Published:** 2012-03-05

**Authors:** Qinghao Xu, Beverly Chou, Bethany Fitzsimmons, Atsushi Miyanohara, Veronica Shubayev, Camila Santucci, Michael Hefferan, Martin Marsala, Xiao-Ying Hua

**Affiliations:** 1 Department of Anesthesiology, University of California San Diego, San Diego, California, United States of America; 2 School of Medicine, University of California San Diego, San Diego, California, United States of America; 3 Institute of Neurobiology, Slovak Academy of Sciences, Košice, Slovakia; University of Florida, United States of America

## Abstract

We report here in adult rat viral vector mediate-gene knockdown in the primary sensory neurons and the associated cellular and behavior consequences. Self-complementary adeno-associated virus serotype 5 (AAV5) was constructed to express green fluorescent protein (GFP) and a small interfering RNA (siRNA) targeting mammalian target of rapamycin (mTOR). The AAV vectors were injected via an intrathecal catheter. We observed profound GFP expression in lumbar DRG neurons beginning at 2-week post-injection. Of those neurons, over 85% were large to medium-diameter and co-labeled with NF200, a marker for myelinated fibers. Western blotting of mTOR revealed an 80% reduction in the lumbar DRGs (L4–L6) of rats treated with the active siRNA vectors compared to the control siRNA vector. Gene knockdown became apparent as early as 7-day post-injection and lasted for at least 5 weeks. Importantly, mTOR knockdown occurred in large (NF200) and small-diameter neurons (nociceptors). The viral administration induced an increase of Iba1 immunoreactivity in the DRGs, which was likely attributed to the expression of GFP but not siRNA. Rats with mTOR knockdown in DRG neurons showed normal general behavior and unaltered responses to noxious stimuli. In conclusion, intrathecal AAV5 is a highly efficient vehicle to deliver siRNA and generate gene knockdown in DRG neurons. This will be valuable for both basic research and clinic intervention of diseases involving primary sensory neurons.

## Introduction

Dorsal root ganglia (DRG) harbor the cell bodies of primary sensory neurons, which send afferent axons and convey sensory information from the periphery to the spinal cord. Abnormal gene expression in primary sensory neurons is implicated in the hyperpathia following nerve and tissue injury. Thus, in chronic pain conditions, a drastic change in the expression of a variety of DRG genes has been noted, including increased expression of sodium channels [1] and the α2δ1 subunit of voltage-gated calcium channels [2]–[3], which are thought to contribute to the hyperexcitability of DRG neurons and the associated hyperalgesia and allodynia. In addition, receptors to cytokines, chemokines and growth factors such as TNF, bradykinin, NGF and catecholamines are increased following nerve injury [4]–[7]. Antagonizing these injury-induced gene changes in DRG neurons can prevent trophic changes and alleviate facilitated pain states. Thus, selective gene knockdown in DRG neurons can be achieved by intrathecal (IT) application of antisense oligodeoxynucleotides (oligos) or siRNAs. Although antisense oligos directed against some pro-nociceptive molecules in DRG (e.g. Nav1.3, Nav1.8) displayed analgesia [8]–[9], the utility of antisense oligos and synthetic siRNA are limited by several factors including toxicity and short-lasting effect [10].

Alternatively, siRNA can be derived from a short hairpin precursor that is expressed from a viral vector [11]. Recently it has been reported that several serotypes of adeno-associated virus (AAV) are efficient in transducing DRG neurons in rodents [12]–[18]. However, the transduction efficiency and tropism in the DRG vary considerably, depending upon the routes of administration, animal species and viral serotypes. AAV5 vectors directly injected into rat DRGs resulted in transduction in up to 90% of the neurons, including most nociceptors [13]. In contrast, following IT injection in mouse, the same serotype targeted large-diameter DRG neurons, while excluding the isolectin-B4 (IB4)–binding, non-peptidergic nociceptors [12]. AAV6 transduces both neurons and satellite cells in rats following direct DRG injection [13] but preferentially transduces neurons following mouse sciatic nerve or IT injection [14]. Understanding the vector tropisms is important for studies aiming to target certain subsets of DRG neurons.

The *in vivo* efficacy of AAV-mediated RNA interference in the nervous system in general has been extensively studied but information regarding the DRG is still limited. A recent paper reported that AAV5 encoding a short-hairpin RNA produced a significant loss of neuropilin2 mRNA in the rat DRG following a direct injection [19]. While the result is interesting, neuropilin2 is not expressed in all DRG neuron populations. It is therefore difficult to determine the cell types that can be targeted by AAV-encoded siRNA. Further, direct injection of DRG requires an invasive surgical procedure that is time-consuming and may itself cause inflammation and pain. Intrathecal administration as a less invasive approach produces satisfactory viral transduction but the capacity of IT vector to deliver siRNA has not been evaluated. In the current study, we aimed to investigate: 1) the transduction efficacy and tropism of an AAV5 vector in rat DRG and spinal cord after an intrathecal delivery, and 2) the efficiency of gene expression knockdown in rat DRG by an siRNA when delivered by the AAV5 vector. We choose mTOR as the target gene, for its ubiquitous expression in DRG neurons and for its reported role in peripheral nociception [20].

## Results

### GFP expression in the DRG and spinal cord following intrathecal AAV5 administration

**Figure 1:**
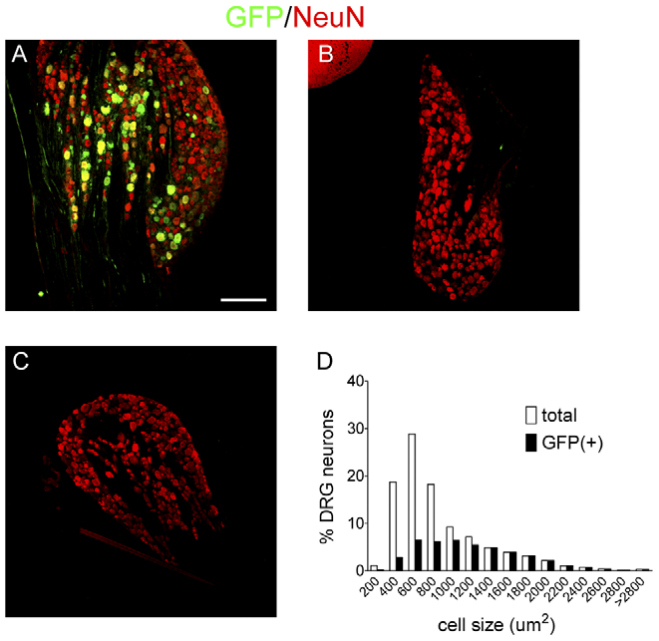
Expression of GFP and NeuN in rat DRG following intrathecal vector injection. A,B and C) GFP (green) and NeuN (red) were co-labeled in A) lumbar, B) thoracic and C) cervical DRG at 2 week-post vector injection. D) Bar graph represents size distribution of DRG neurons with and without GFP labeling. A total of 3781 neurons from the L4, L5 and L6 DRGs of 3 animals were included. Scale bar, 250 µm.

**Figure 2:**
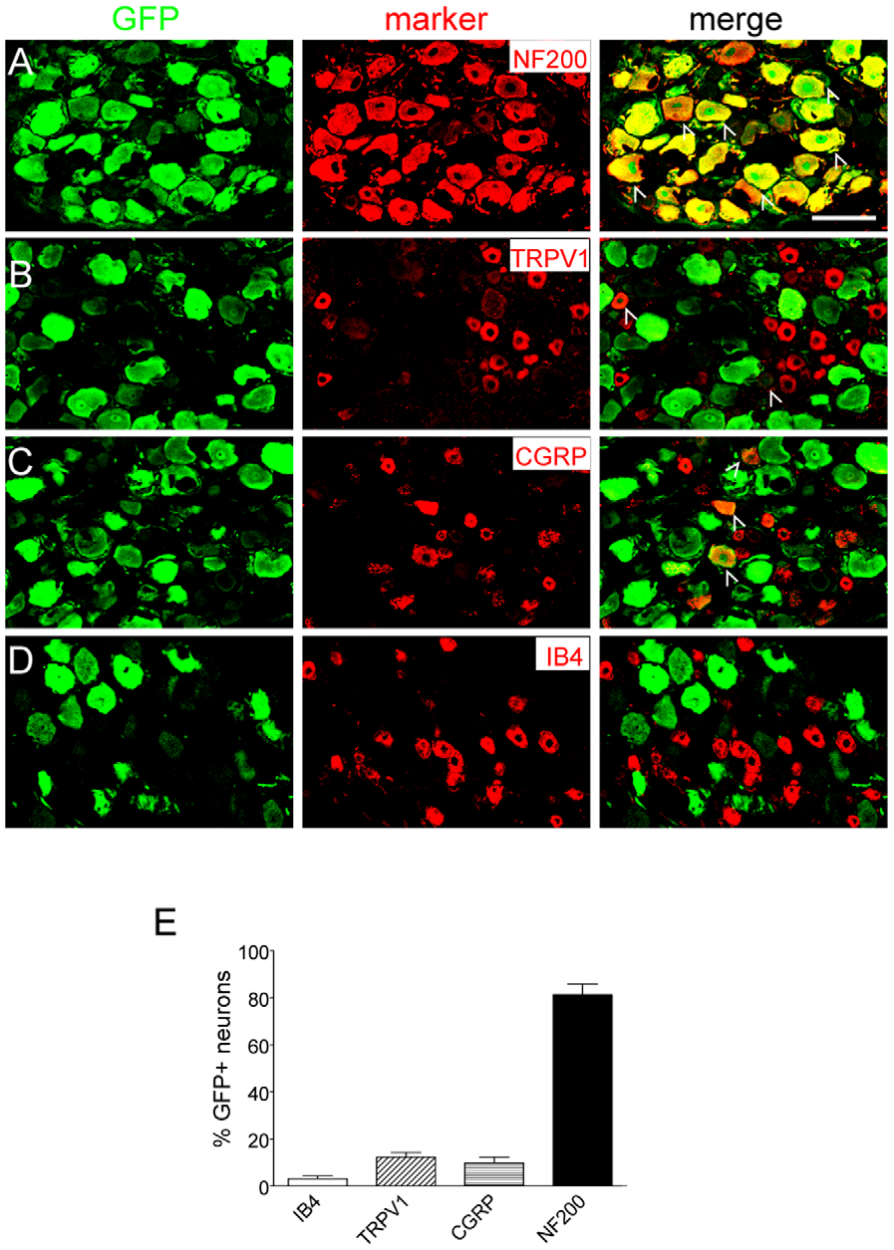
Expression of GFP in different populations of neurons in the lumbar DRG. A,B C and D) GFP (green) was co-labeled with A) NF200, B) TRPV1, C) CGRP and D) IB4 (red) at two week following vector administration. Arrowheads indicate co-localization of GFP with the respective cell marker. E) Bar graph represents percentage of GFP-positive neurons co-labeled with the aforementioned markers. The data are presented as mean ± SEM of 3 rats. Scale bar, 100 µm.

To study the transduction efficacy and tropism of AAV5 in rats, we first constructed a vector encoding GFP driven by a cytomegalovirus (CMV) promoter. Ten microliters of virus (10^11^ viral particles) was administered in adult rats through an intrathecal catheter, with the tip ending at the spinal level of L3–L4. The GFP expression began to be visualized in the DRG and spinal cord at 1 week following the vector administration, however the intensity of GFP immunoreactivity at this time point was very weak (not shown), and the distribution pattern was not analyzed. A profound GFP expression was observed after 2 weeks in the lumbar DRG neurons (Figure 1a), while in the thoracic and cervical DRGs GFP was not detectable (figure 1b and c). We have analyzed a total of 3781 neurons in the L4, L5 and L6 DRGs from three rats, 44% (1672) of those neurons were GFP-positive. Interestingly, the GFP-labeled neurons were predominantly of large to medium size (figure 1d). Out of all the GFP-positive profiles in the DRG, 38% were large (>1200 µm^2^), 41% were medium (>600 µm^2^and <1200 µm^2^) and only 21% were small (<600 µm^2^). Conversely, out of all the neuronal profiles, GFP was observed in 100%, 52% and 20% in large, medium and small profiles respectively. Double immunofluorescence study revealed that over 80% of the GFP-positive neurons were co-labeled with NF200, a marker for myelinated fibers (figure 2a and e). A small portion of transduced neurons are co-labeled with nociceptor markers such as TRPV1, CGRP and IB4 (figure 2b–e).

**Figure 3:**
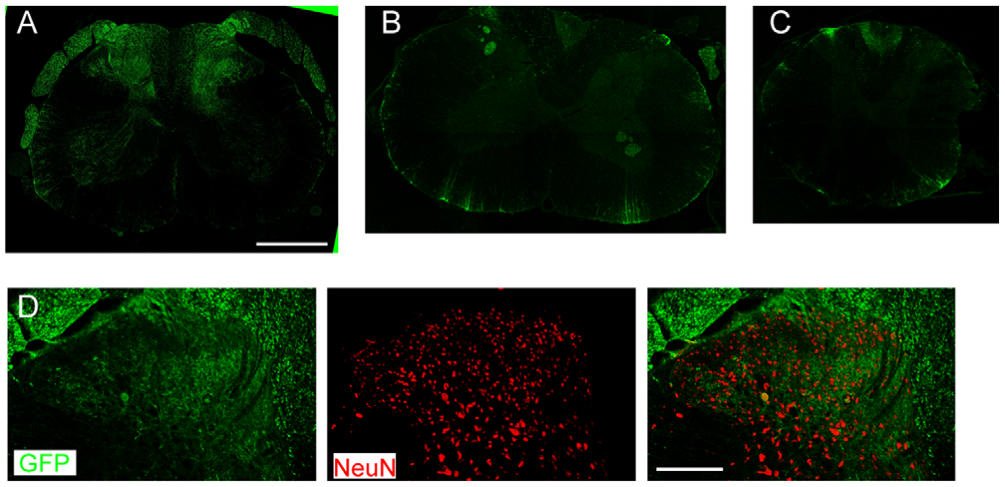
Expression of GFP in the spinal cord following intrathecal vector injection. A,B and C) GFP (green) expression in A) lumbar, B) cervical and C) thoracic spinal cord at 2 week-post intrathecal vector injection. D) Co-labeling of GFP (green) and NeuN (red) in the lumbar spinal cord dorsal horn. Scale bars, A–C, 1000 µm; D, 250 um.

In the spinal cord, GFP was detected in abundance at the lumbar level (figure 3a), and to a much less degree at the thoracic or cervical level (figure 3b and c). The spinal GFP immunoreactivity was mostly present in the deeper dorsal horn, dorsal columns and nerve roots attached. GFP was also observed in the ventral horn and white matter, although the labeling intensity was much lower (figure 3a–c). In the dorsal horn, the fibrous pattern and minimal co-labeling with spinal cord neuN (figure 3d) suggested the major source of GFP was primary afferents. There was a preferential co-labeling of GFP with NF200 but not IB4 or CGRP in the spinal cord dorsal horn (figure S1). In addition, GFP was observed in the sciatic nerves and the nerve terminals in the plantar skin of the hind paw (figure S2).

### AAV5-mediated knockdown of mTOR expression

**Figure 4:**
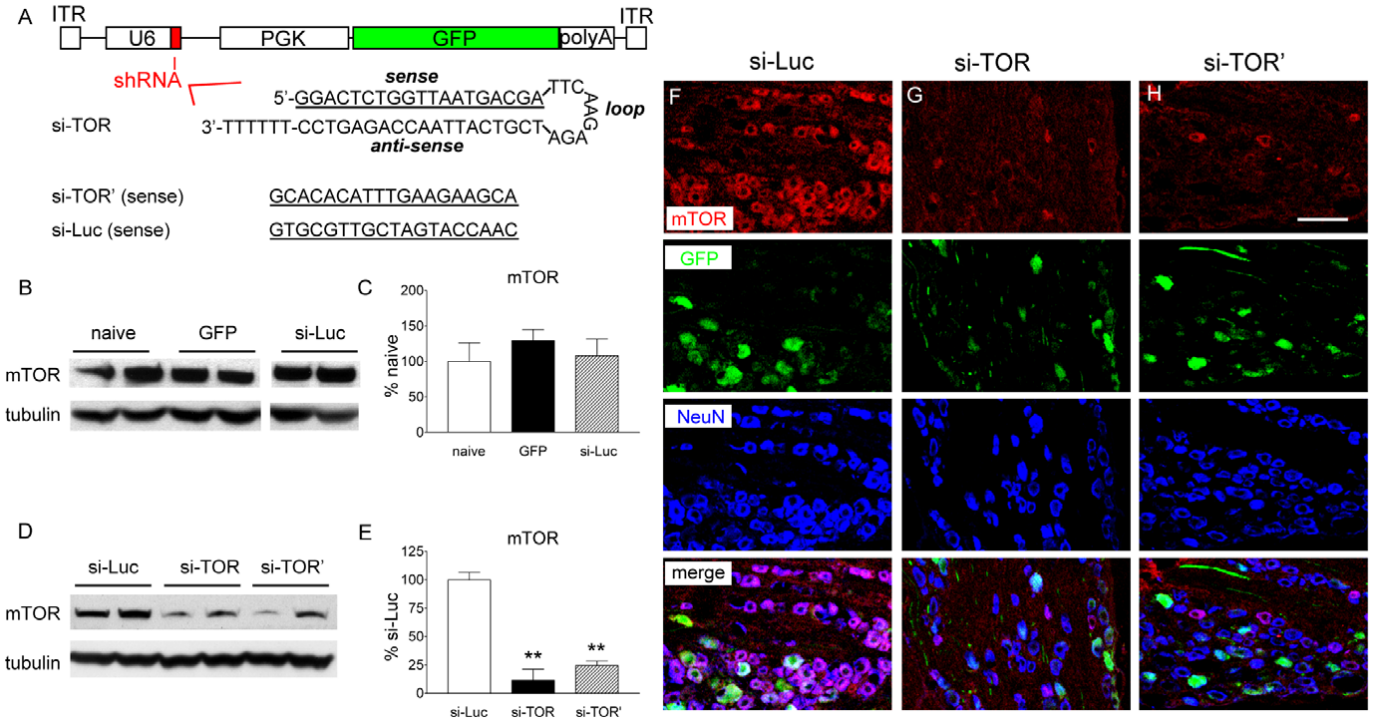
Expression of mTOR in lumbar DRGs following intrathecal vector injection. A) Schematic drawing of the AAV vector that encodes sh-RNA. The sequences of the two active and one control siRNA (si-Luc) are shown. B) Representative Western blots showing levels of mTOR and beta-tubulin in naïve rats or at two week following vector administration in the lumbar DRGs (L4 and L5 DRGs from both sides were pooled). C) Histogram represents the mean mTOR levels in respect to the naive group. The data are presented as mean ± SEM of 4–6 rats per group. D) Representative Western blots showing levels of mTOR and beta-tubulin at five week following vector administration in the lumbar DRGs (L4 and L5 DRGs from both sides were pooled). E) Histogram represents the mean mTOR levels in respect to the control group (si-Luc). The data are presented as mean ± SEM of 4 rats per group. F, G and H) Confocal images of mTOR (red), GFP (green) and NeuN (blue) in L5 DRG at two week following F) si-Luc, G) si-TOR and H) si-TOR' vector administration. Scale bar, 100 µm. PGK, phosphoglycerate kinase promoter; ITR, inverted terminal repeat; U6, U6 promoter.

A similar preference for large-diameter DRG neurons by AAV8 and AAV9 was observed in our pilot study, while the transduction efficacy of AAV5 was evidently the most consistent and outstanding among the three AAVs. We therefore selected AAV5 in the following siRNA studies. Two siRNAs targeting mTOR (si-TOR and si-TOR') and one control siRNA targeting luciferase (si-Luc) were constructed as short-hairpin RNAs downstream of a U6 promoter (figure 4a). The vectors also encoded a GFP gene driven by a phosphoglycerate kinase (PGK) promoter. GFP-alone vector or si-Luc vector treatment did not alter the mTOR level in the lumbar DRGs, compared to the naïve control (figure 4b and c). However, a significant reduction of mTOR was observed in the lumbar DRG homogenates in rats treated with either of the mTOR siRNA vectors at two week following the IT administration (figure 4d and e). The two vectors exhibited similar potency and induced a comparable degree of mTOR knockdown (figure 4dand e, a reduction of 88% for si-TOR and 76% for si-TOR', compared to si-Luc). Confocal immunohistochemistry revealed that the pattern of GFP expression driven by the PGK promoter is similar to that driven by the CMV promoter (the siRNA-less vector). Strong GFP expression is predominantly present in large to medium-diameter neurons and preferentially co-localized with NF200 (figure S3). mTOR labeling is intact in rats treated with the control si-Luc vector and present in most if not all neurons (fig. 4f). As a result, extensive co-labeling of mTOR and GFP was observed. The level of mTOR was greatly reduced in DRG neurons from rats received si-TOR or si-TOR' vectors and the co-labeling of mTOR with GFP was profoundly reduced (fig. 4g and h). The down-regulation of mTOR is not observed in the lumbar spinal cord or DRGs from thoracic or cervical levels (figure S4).

**Figure 5:**
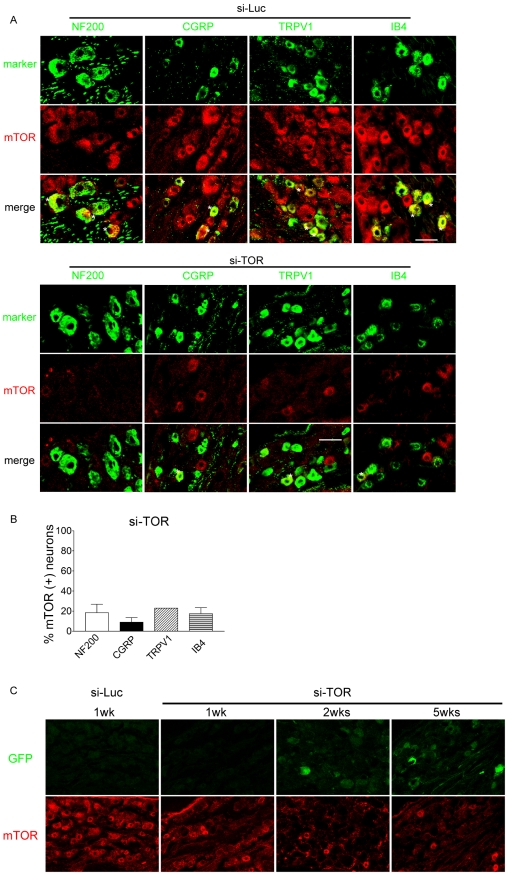
Expression of mTOR in L5 DRG following intrathecal vector administration. A) Confocal images of mTOR (red) co-labeled with NF200, CGRP, TRPV1 or IB4 (green) in L5 DRG 2 weeks following the si-Luc or si-TOR vector injection. Double labeled neurons are indicated with asterisks. B) Bar graph represents percentage of mTOR-positive neurons co-labeled with the aforementioned markers. The data are presented as mean ± SEM of 3–4 rats except TRPV1 represents a single rat. C) GFP (green) and mTOR (red) in L5 DRG at 1 week following the si-Luc vector and 1, 2 and 5 weeks following the si-TOR vector injection. Scale bars, A, 50 µm and C, 100 µm.

Interestingly but unexpectedly, the knockdown of mTOR was not confined to GFP-positive neurons (figure 4g and h). A significant portion of nociceptors (i.e. small diameter, type B ganglion neurons), which did not express detectable GFP, also exhibited reduced mTOR labeling after the si-TOR treatment (figure 5a and b). This prompted us to further examine the time course of mTOR knockdown in a correlation with the expression of GFP. We found that unlike GFP, which was detectable beginning at 2 weeks following the vector injection, the down-regulation of mTOR became apparent as early as 1 week (figure 5c). At this time point, GFP expression was significantly lower. This observation provides important information suggesting that though the efficacy of viral transduction is often evaluated by GFP expression, it may underestimate the RNA interference effects. The siRNA-mediated knockdown lasted for at least 5 weeks, the longest time period examined in this study (figure 5c).

### Cytotoxicity and behavior consequences following the mTOR expression knockdown

**Figure 6:**
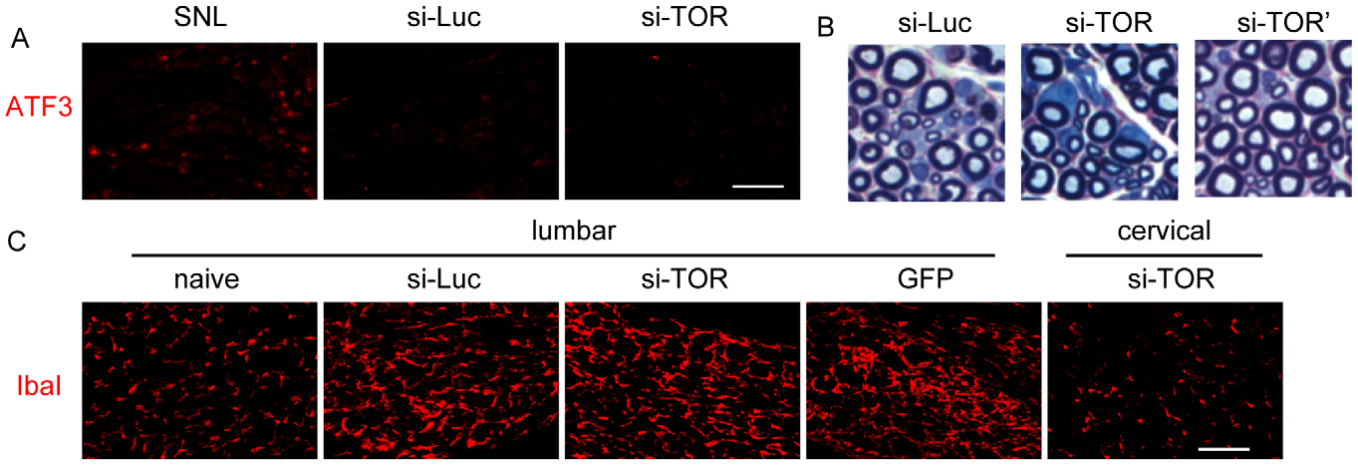
DRG ATF3, Iba1 immunohistochemistry and sciatic nerve myelin staining following intrathecal vector injection. A) Expression of ATF3 in the L5 DRGs in rats treated with spinal nerve ligation (SNL, 2 weeks), si-Luc or si-TOR AAV5 vectors (4 weeks). B) Myelin staining of the sciatic nerve from rats treated with si-Luc, si-TOR or si-TOR' vectors (4 weeks). C) Expression of IbaI in the L5 DRGs in a naïve rat or rats received si-Luc, si-TOR or GFP-only vectors. A cervical DRG from a rat treated with si-TOR vector was also shown. Scale bar, 100 µm.

We next assessed the cytotoxicity of the intrathecally administered AAV5 siRNA vectors. In the lumbar DRG where most of the transduction occurred, neurons appeared healthy judged by NeuN immunolabeling (figure 4f–h). We did not observe any expression of ATF3, a marker for neuronal injury (figure 6a), even in neurons with intense GFP expression. There was no visible myelin damage in the sciatic nerves (figure 6b). We did, however, notice an increase of Iba1 immunoreactivity in the lumbar DRGs (figure 6c), which could indicate the activation of microglia or invasion of macrophages. There was a similar degree of Iba1 increase after the three siRNA vectors or the siRNA-less vector (CMV-GFP), suggesting this phenomenon was unlikely due to mTOR knockdown or the expression of vector-derived small RNAs. The Iba1 expression in the cervical DRG (figure 6c) or the spinal cord (data not shown) was not elevated.

**Figure 7:**
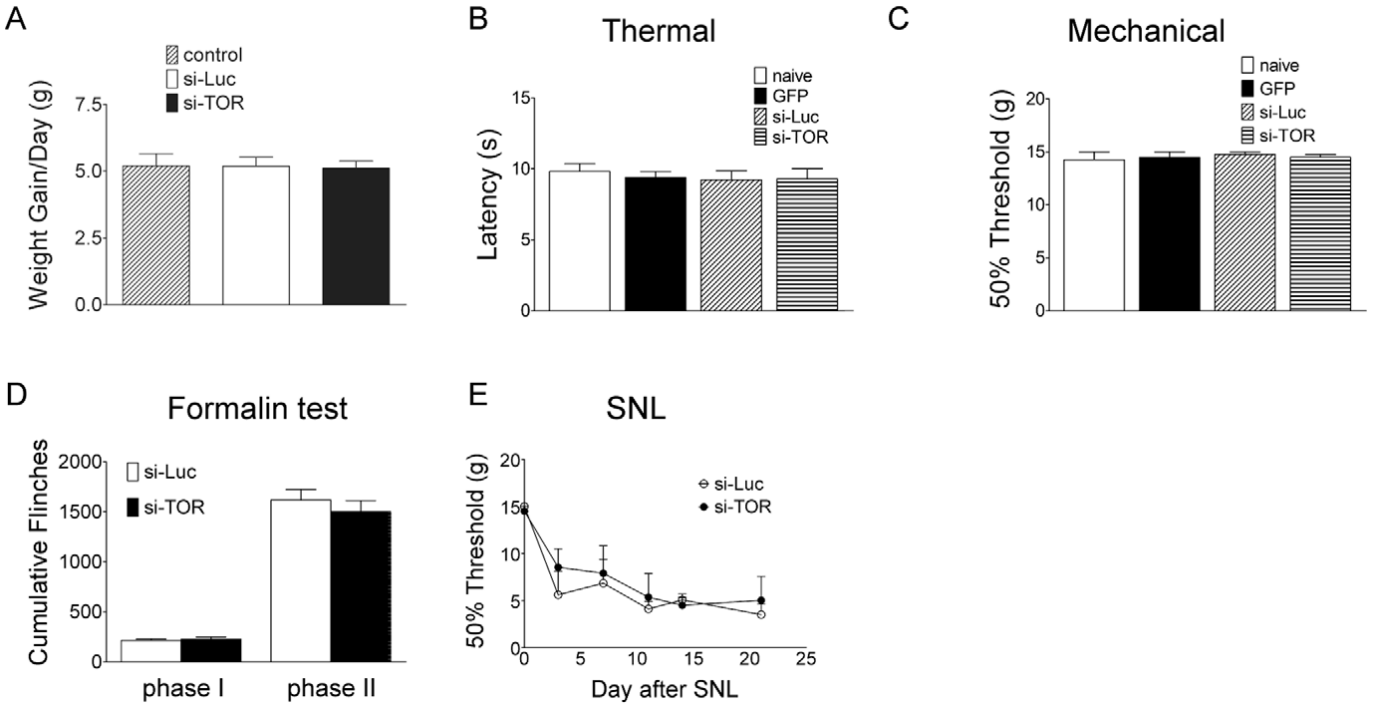
Weight gain and nociceptive behaviors following the siRNA vectors. A) Daily weight gain of rats after receiving no vector (control), si-Luc or si-TOR vector. The mean weight gain in a two week period following vector injection is shown. B) Thermal and C) mechanical thresholds for naïve rats and rats treated with GFP, si-Luc or si-TOR vector for two weeks. D) Total number of flinches in phase I (1–9 min) and II (10–60 min) following intraplantar formalin (2.5%, 50 µL) injection. Rats received si-Luc or si-TOR vector two weeks before the formalin tests. E) Mechanical thresholds of the ipsilateral paw following unilateral L5 and L6 spinal nerve ligation. Rats received si-Luc or si-TOR vector two weeks before the SNL surgery. The data are presented as mean ± SEM of 6–8 rats per group.

We further assessed the changes in pain behaviors following the mTOR knockdown in the lumbar DRGs. Rats treated with the siRNA vectors exhibited similar weight gain as the age-matched animals that were catheterized but did not receive an AAV vector (figure 7a). Compared to the naïve rats or GFP vector treated rats, si-Luc and si-TOR group had comparable baseline thermal and tactile pain thresholds (figure 7b and c). Rats developed similar degree of flinching behavior in the formalin model (figure 7d) and allodynia in the spinal nerve ligation neuropathic pain model (figure 7e) in the si-Luc and si-TOR groups.

## Discussion

Gene silencing in the primary sensory neurons is desirable for basic research such as neuronal development, nerve regeneration and mechanisms of pain, and, now, for development of potential therapeutics. Gene expression knockdown in DRG neurons can be achieved by intrathecal (IT) delivery of synthetic siRNA or antisense oligodeoxynucleotides. However, the knockdown is usually transient and often associated with toxicity. In the current study, we characterized an alternative approach, AAV-encoded siRNA, to produce inhibition of gene expression in DRG neurons. We demonstrated that AAV5, when injected intrathecally, was highly efficient in transducing DRG neurons and establishing gene expression knockdown. We further demonstrated that the knockdown targeted all neuron populations and was associated with minimal cytotoxicity.

The transduction rate of AAV5 in DRG neurons was estimated to be 50% using GFP as a marker. AAV5 appeared to favor NF200-positive, large to medium-diameter DRG neurons in our study. Importantly, the nociceptors, especially the IB4-positive population, were found to express low level or no GFP. This transduction profile is similar to a study using mice, where AAV5 was administered intrathecally by lumbar puncture [12], though in the case of direct injection of AAV5 to DRG there was GFP expression in nociceptors including IB4-positive, non-peptidergic neurons [13].

In the spinal cord sections, GFP was mainly detected in the dorsal column, deep dorsal horn and dorsal roots. This distribution was consistent with the prominent GFP labeling in the NF200-positive DRG neurons, which give rise to large-diameter, myelinated primary afferents and the transport to the distal terminals. The lack of GFP labeling in the superficial layers paralleled the very modest expression of GFP in nociceptors. Importantly, there was almost no cell body labeling in the dorsal horn, suggesting that AAV5 vector either does not penetrate well into the gray matter or was not taken up by non-afferent neurons. It has been hypothesized that the viral vector may enter the DRG neurons through dorsal roots, which are accessible to intrathecally administered agents [10].

In some rats, we did observe lower intensity GFP labeling in the ventral horn and white matter. Similar to dorsal horn labeling, ventral horn GFP was predominantly associated with nerve fibers. Occasionally we could see ventral horn cell bodies, presumably motor neurons labeled with GFP in the sacral region. The GFP labeling in the white matter was almost invariably associated with GFAP (not shown), suggesting it was of astrocyte origin.

AAV5 was chosen over other serotypes (AAV2, 6, 8 and 9) for superior transduction efficacy in the DRG as evidenced by GFP expression, a finding consistent with a previous report [13]. In our preliminary study, IT AAV2 and 6 produced no detectable GFP expression, neither in the DRG nor in spinal cord, although a previous study reported superb transduction in mouse DRG by IT AAV6 [14]. AAV8 and AAV9 produced substantial amount of GFP in some DRG neurons however the diffusion was limited compared to AAV5. We think low dose was a possible reason to explain why those vectors were outperformed by AAV5 in our preliminary study. Interestingly, different AAV serotypes seem to target different populations of neurons even with the same route of delivery. Intrathecal AAV6 preferentially transduced nociceptors [14] while AAV9 produced considerable labeling in motor neurons [21]–[22]. The mechanism behind these preferences remains to be deciphered.

An important observation was that GFP expression was largely confined to the lumbar area. Few cells from the cervical and thoracic DRGs were labeled with detectable GFP. Indeed, there was no GFP expression in the cervical or thoracic dorsal horn. The weak GFP expression that was restricted to the dorsal column likely comes from the proprioceptive primary afferent neurons at the lumbar level. This spatial localization of viral transduction reflects the lack of prominent cerebrospinal fluid (CSF) movement pattern in the intrathecal space. Indeed, many studies have shown that the circulation of the CSF is limited and the diffusion of large particles (e.g. AAV) in the CSF is relatively slow [23]–[24]. The spatially limited transduction is of particular advantage where targeting of only the lumbar DRGs is desired. The spatial confinement of transduction, as is the distribution of most intrathecally delivered agents, can be manipulated by changing the positioning of the catheter tip and the volume of viral vector dosing. Smaller volumes would likely be useful under these circumstances for targeting of a yet fewer number of DRGs. For transduction of a specific ganglion, a direct injection may be more appropriate, but that brings out the issue regarding the likelihood of injury.

mTOR is distributed in all populations of DRG neurons [25], where it has been implicated in peripheral nociception [20]. In the present study, we used AAV5-encoded shRNA to generate a knockdown of mTOR gene expression that was confined to DRG neurons. The gene knockdown was selective, long-lasting and segmentally localized (only observed in the lumbar DRGs). At 5 weeks following the vector administration, the mTOR protein was reduced to 20% of the control level. To our surprise, mTOR silencing was observed in many more cells than what would have been predicted from the GFP distribution. Significant mTOR reduction was not only observed in large to medium-diameter neurons, but also in various groups of nociceptors, including IB4-positive nociceptors, which displayed no detectable GFP. This observation indicates AAV5 was able to transduce most if not all groups of DRG neurons. However, the low expression level of GFP in some neuron populations (i.e. nociceptors) might have lead to an underestimation of the vector transduction rate. A number of factors could contribute to the expression disparity of GFP. Higher level of GFP expression in large-diameter neurons may result from higher copies of viral genomes present in the cell, subsequently higher rate of transcription and translation. It was reported that the level of GFP mRNA peaked after 1 week following a direct injection of AAV5 into the DRG, while the level of GFP protein kept increasing throughout a 12-week period [13]. It is possible that, within the time frame of our experiment (5 weeks), GFP might not have reached detectable level in nociceptors. For more accurate assessment of viral transduction, other means such as *in situ* hybridization shall be considered.

In previous reports, vector shRNA-mediated knockdown and the expression of a reporter gene (e.g. GFP) usually correlate well in their spatial distribution [26]–[27]. However, current results suggest that they should be treated as two separate events which do not necessarily parallel each other. The required level of siRNA to induce RNA interference is likely different from that of GFP mRNA to mediate detectable GFP expression. Moreover, the siRNA was presumably transcribed by RNA polymerase III, which recognizes the U6 promoter. The transcription of GFP mRNA was driven by an RNA polymerase II promoter. It is conceivable that the two types of RNA polymerases work independently and the siRNA and mRNA were produced at different rates. These hypotheses may be addressed in future experiments.

Self-complementary (sc) AAV vectors bypass the rate-limiting step of second-strand synthesis. This type of AAV is known to mediate faster onset of transgene expression [28]–[29]. GFP was detected in retinal pigment epithelium as early as day 1 following trans-cornea sub-retinal injection of a sc-AAV5 vector [30]. Although we did not evaluate GFP expression or mTOR knockdown at such an early stage, we found that at 1 week following vector injection, there was strong mTOR downregulation in the target tissues. The results indicate IT AAV-mediate RNAi effects are well within a time frame that is suitable for clinical intervention of diseases.

mTOR is reportedly involved in many cellular processes, notably cell replication and differentiation. mTOR inhibitors are currently under development for potential anti-cancer drugs. It is apparent that knocking down mTOR has little effects on the survival of primary sensory neurons, possibly due to the fact that these neurons are terminally differentiated. Transduced DRG neurons exhibited normal, healthy morphology which was comparable to naïve tissues, regardless of the level of mTOR expression. No signs of neuronal damage such as ATF3 induction or myelin loss have been observed. The data also argue for a lack of toxicity from vector-derived siRNA in DRG neurons, which is consistent with a recent report [19]. An AAV5 vector encoding a siRNA was directly injected into the DRG and no detectable cytotoxicity was reported.

An increase in DRG Iba1 immunoreactivity was the only sign of vector-induced adverse side-effects that we could detect. In many cases, increased Iba1 is viewed as an indicator for microglia/macrophage activation. It is unlikely that the glia activation was directly related to the vector-mediated RNAi effects, because a comparable degree of Iba1 upregulation was observed following an “RNA-less” AAV5 vector that encodes GFP only. There is, however, a possibility that the increased Iba1 is attributable to GFP, or alternatively to the viral capsid. To note, the Iba1 increase was present only in the lumbar DRGs. In thoracic or cervical DRGs, where viral transduction was negative, there was no Iba1 increase. It would be interesting to investigate in future studies if a “GFP-less” AAV5 vector or other serotypes elicit the same reaction. Despite the apparent Iba1 increase in the lumbar DRGs, AAV5-treated rats displayed overall good health, normal weight gain and no detectable signs of stress. Baseline pain thresholds, a sensitive measure of function, were unaltered suggesting no abnormities.

mTOR has been implicated in nociceptive signal transmission, both at peripheral sites and spinal dorsal horn [20], [31]–[34]. A previous study [20] showed that peripheral inhibition of mTOR by intraplantar rapamycin decreases the thermal sensitivity of a subset of A-nociceptors in non-injured rats, and local rapamycin treatment attenuated mechanical hypersensitivity in the spared nerve injury (SNI) neuropathic pain model. However, we did not observe any changes on pain thresholds in rats with DRG mTOR knockdown, neither in the acute thermal or mechanical tests nor the two chronic pain models. A significant reduction of mTOR protein in lumbar DRGs was confirmed by both Western blotting and immunohistochemistry. We also observed a decrease of phospho-S6 protein (data not shown), suggestive of an inhibition of mTOR enzymatic activity, in the treated lumbar DRGs. It is known that in addition to DRG, mTOR is expressed extensively in spinal dorsal horn neurons including the NK1 projection neurons which are essential in pain signal transduction (Xu et al 2011). Since mTOR expression in spinal cord is intact in the AAV-treated animals (see figure S4), given the observed spinal pharmacology of mTOR, we suggest that mTOR function in primary sensory neurons (as knocked out selectively with IT–AAV5) is not essential for nociception. It should be noted that other signaling pathways, such as mitogen-activated protein kinase (MAPK) pathways exist in the primary sensory neurons that may also play important roles. These alternative pathways may have undergone adaptive changes to compensate for a chronic knockdown of mTOR and a normal pain behavior was preserved. It is interesting to note that IT antisense oligo knockdown of a p38 MAPK isoform (p38-β) indeed diminished the hyperalgesic pain state [35]. Those studies do not permit assessment of whether the relevant site of p38 knock down was spinal or DRG. Further experiments could indeed assess the role of DRG-p38-β by IT-AAV5-mediated transduction.

In conclusion, the current study showed that intrathecal administration of AAV5 is a highly efficient and reliable paradigm for delivering small RNAs and generating gene knockdowns in the primary afferent neurons. The majority populations of DRG neurons were targeted by the RNAi effect, regardless of the expression level of a reporter gene, GFP. Intrathecal AAV5 shall be exploited further for the studies and treatments of diseases affecting DRG neurons. Further development of this approach will require assessment of the distribution properties in large animal model (e.g. dog) [36]–[37] to define safety and efficacy.

## Materials and Methods

### Ethics Statement

All experiment protocols (S00148R) were approved by the University of California, San Diego, Institutional Animal Care and Use Committee in accordance with the Guide for the Care and Use of Laboratory Animals (National Institutes of Health publication 85–23, Bethesda, MD, USA).

### Animals

Male Sprague–Dawley rats (200–250 g; Harlan, Indianapolis, IN, USA) were housed in a light-controlled room (light from 07:00–19:00 h) with free access to food and water. Surgical procedures and testing occurred during the light cycle. Measures were taken to minimize the pain and discomfort of the experimental animals.

### Construction and preparation of viral vectors

siRNA targeting the rat mTOR mRNA (NM_019906) were selected by the ‘siRNA design program’ (http://jura.wi.mit.edu/siRNAext/) from the Whitehead Institute for Biomedical Research [38]. A spacer (TTCAAGAGA) was inserted into the antisense and sense sequences to generate a “stem-loop” structure [11]. DNA oligomers were synthesized according to the “stem-loop” sequences (Integrated DNA Technologies, Coralville, Iowa), annealed and cloned into an AAV plasmid. The oligomers were placed downstream of a U6 promoter and the insertion was confirmed by sequencing. The same AAV plasmid also encoded green fluorescent protein driven by a phosphoglycerate kinase promoter.

To produce self-complementary double-strand DNA genome AAV vectors (scAAVs), a 20 base pair sequence was deleted in the terminal resolution site (TRS) in one of the two ITRs in the pAAV plasmid [29], [39]. Helper virus-free scAAV5 vectors were produced by transient transfection of HEK293T cells with the vector plasmid, pRep2-Cap5 and pAd-Helper plasmid [40]. Plasmid pRep2-Cap5 was constructed at the UCSD Vector Core by sub-cloning of the Cap gene from pXYZ5 (obtained from Dr. Snyder, U. Florida [41]). Cell lysates prepared at 72 hr after transfection were treated with benzonase and viruses were pelleted through 25% sucrose-cushion ultracentrifugation. The pellets were resuspended and the viruses were further purified through anion-exchange column chromatography (Q-Sepharose, GE Health Science) followed by concentration through 25% sucrose-cushion ultracentrifugation. The final pellets were resuspended with a solution consisting of 10 mM Tris-HCl, pH7.9, 1 mM MgCl_2_ and 3% sucrose. Virus titers were determined by measuring the genome copies by real-time Q-PCR.

### Intrathecal viral vector administration

The implantation of intrathecal catheter in rats has been previously described [42]–[43]. AAV5 vectors were prepared to a concentration of 1×10^13^ viral particles/mL. Vectors were delivered in 10 µL followed by 10 µL of saline through the intrathecal catheter 24 hr following the implantation. Catheters were removed 72 hr following the vector injection.

### Western blotting (WB)

Five weeks following the vector injection, rats were deeply anesthetized by isoflurane and decapitated. Lumbar spinal cord dorsal quadrants and DRGs from the L4 and L5 levels were harvested in extraction buffer [50 mM Tris buffer, pH 7.4, containing 0.5% Triton X-100, 150 mM NaCl, 1 mM EDTA, 3% SDS, protease inhibitor cocktail (1∶100, P8340, Sigma) and phosphatase inhibitor cocktail I and II (1∶100, P2850 and P5726, Sigma)]. Samples were sonicated, centrifuged at 14,500 rpm for 15 min and the supernatants were subject to sodium dodecyl sulfate polyacrylamide gel electrophoresis (4–12% Bis-Tris gel, Invitrogen, Carlsbad, CA, USA). Samples were transferred to nitrocellulose membranes and incubated with the primary antibody (mTOR, 1∶1000, Cat. No. 2972, 1∶1000, Cell Signaling Technology, Danvers, MA) overnight at 4°C. After washing, membranes were probed with appropriate Horseradish Peroxidase conjugated secondary antibody diluted at 1∶10,000. Membranes were then incubated with SuperSignal Chemiluminescent Substrate reagents (Pierce, Rockford, IL, USA) and the luminescent signal was exposed to film and developed for further scanning and quantification. The membranes were stripped with a Re-Blot western blot recycling kit (Chemicon, Temecula, CA, USA) and re-probed with β-tubulin (1∶50,000, Sigma, St. Louis, MO). The optical density (OD) of immunoreactive bands was quantified using ImageQuant software (Molecular Dynamics, Sunnyvale, CA, USA).

### Immunohistochemistry (IHC)

Animals were terminally anesthetized with Euthasol (0.5 mL, Virbac Animal Health, Inc. Fort Worth, TX, USA) and transcardially perfused with saline followed by 4% paraformaldehyde in PBS. Tissues were dissected and post-fixed in the same fixative for 2 hr at 4°C. The tissues were then transferred to 30% sucrose at 4°C for 72 hr. Spinal cord (30 µm), sciatic nerve (30 µm), skin (30 µm) and DRG (10 µm) sections were cut on a Leica cryostat (CM1800) and mounted on superfrost plus slides (12-550-15, Fisher Scientific, Pittsburgh, PA, USA) before proceeding to IHC. Non-specific binding was blocked by incubation in 3% normal goat serum or 3% normal donkey serum (vector laboratories, Malden, MA, USA) in PBS with 0.3% Triton X-100 followed by incubation with the primary antibodies overnight. The antibodies used were: rabbit anti-mTOR (1∶500, 2972, Cell Signaling Technology, Danvers, MA), rabbit anti-GFP (1∶1000, A6455, Invitrogen, Carlsbad, CA), rabbit anti-ATF3 (1∶1000, sc-188, Santa Cruz Biotechnologies; Santa Cruz, CA), mouse anti-NeuN (1∶500; MAB377, Millipore, Temecula, CA), mouse anti-glial fibrillary acidic protein (GFAP, 1∶1000, MAB360, Millipore, Temecula, CA), rabbit anti-Iba-1 (1∶1000, 019-19741, Wako, Richmond, VA); goat anti-TRPV1 (1∶500, sc-12498, Santa Cruz Biotechnologies; Santa Cruz, CA), goat anti-CGRP (1∶500, ab36001, Abcam, Cambridge, MA), mouse anti-NF200 (1∶1000, N0142, Sigma, St. Louis, MO), Alexa647-IB4 (1∶1000, I32450, Invitrogen, Carlsbad, CA). Slides were washed in PBS and incubated in appropriate fluorescent secondary antibodies (Invitrogen, Carlsbad, CA). Some tissues were counterstained with TO-PRO-3 (1∶1000, Molecular Probes). Coverslips were mounted with ProLong antifade medium (Molecular Probes, Eugene, OR, USA). Non-specific labeling was determined by excluding the primary antibodies.

### Confocal microscopy and quantification

Confocal images were acquired by a Leica TCS SP5 confocal system at fixed settings using a 10× or 20× objective at a digital size of 1024×1024 pixels. In case of multiple-labeled IHC, images of each channel were acquired by sequential scanning. NIH Image J was used to quantify the colocalization of different cell markers and GFP or mTOR by a researcher who was blind to the treatment. Cell profiles were manually encircled and the intensity of each color channel was recorded. The threshold for positive labeling was arbitrarily set as 2 folds of the average intensity on the control IHC tissue (primary antibody omitted). For GFP labeling, the threshold was determined on naïve tissue with no viral vector injection. The images were adjusted with Adobe Photoshop software to enhance clarity.

### Myelin staining of sciatic nerves

Plastic-embedded 1 µm-thick nerve sections were used to analyze myelin neuropathology, as previously described [44]. Anesthetized rats were perfused with 4% paraformaldehyde. The sciatic nerves were isolated and fixed in 2.5% glutaraldehyde in 0.1 M phosphate buffer, post-fixed in 1% aqueous osmium tetroxide, dehydrated and embedded in araldite resin. Sections were cut with a glass knife on an automated Lieca RM2065 microtome and stained with Methylene blue Azure II for light microscopic examination.

### Behavior tests

All behavioral measurements were made by observers blinded to the treatment groups.

### Baseline thermal and mechanical threshold

Paw withdrawal latency to a thermal stimulus was measured by a Hargreaves type device [45]. The hind paw was stimulated by a radiant heat. A withdrawal of the paw from the heat source would terminate the stimulus and the latency was recorded automatically. Mechanically induced response in the hind paws was determined by the “up-down” method using von Frey filaments [46].

### Formalin-induced flinching behavior

Formalin-induced flinching was quantified by an automated system [47]. A metal band was glued to the plantar surface of the left hind paw. The dorsal surface was injected with formalin solution (2.5% in physiological saline, 50 µL) by a 30-gauge needle. Rats were placed into the test chamber immediately and the number of flinches of the injected paw was counted automatically. The data are expressed as total number of flinches observed during phase 1 (0–9 min) and phase 2 (10–60 min).

### Spinal nerve ligation

Rats were anesthetized with isoflurane and the L5 transverse process on the left side was partially removed to expose the L5 and L6 spinal nerve. A 6-0 silk tight ligation was made on the L5 and L6 spinal nerve [48]. Rats were allowed to recover for three days before behavior assessment.

### Statistics

The data are presented as the mean ± S.E.M. One-way ANOVA followed by a Tukey *post-hoc* test was used when more than two groups of data were compared; student's *t-*test was used when two groups were compared.

## Supporting Information

Figure S1 Co-labeling of GFP (green) with IB4, CGRP or NF200 (red) in the lumbar spinal cord at 2 week-post intrathecal AAV5 vector injection. Scale bar, 250 µm.
(TIF)

Figure S2 Expression of GFP (green) and NF200 (red) in (A and B) the glabrous skin of the hind paw and (C) sciatic nerves. ep, epidermis; d, dermis. scale bars, a, 200 µm; B and C, 50 µm.
(TIF)

Figure S3 GFP (green) in lumbar DRG co-labeled with NF200 (red) and IB4 (blue) at two week following si-Luc or si-TOR vector administration. Scale bar, 100 µm.
(TIF)

Figure S4 Expression of mTOR in the lumbar spinal cord and cervical and thoracic DRGs. A) Representative Western blots showing levels of mTOR and beta-tubulin in the lumbar spinal cord dorsal horn at 5 week following vector administration. B) Histogram represents the mean mTOR levels in respect to the control group (si-Luc). C) Confocal images of mTOR in T12 and C4 DRG 2 weeks following the si-TOR vector injection. Scale bar, 100 µm.
(TIF)
