# Supplementary figures and images for: In Vivo Gene Knockdown in Rat Dorsal Root Ganglia Mediated by Self-Complementary Adeno-Associated Virus Serotype 5 Following Intrathecal Delivery
Source: PLoS One. 2012 Mar 5;7(3):e32581. doi: 10.1371/journal.pone.0032581 (PMC3293818; doi:10.1371/journal.pone.0032581)

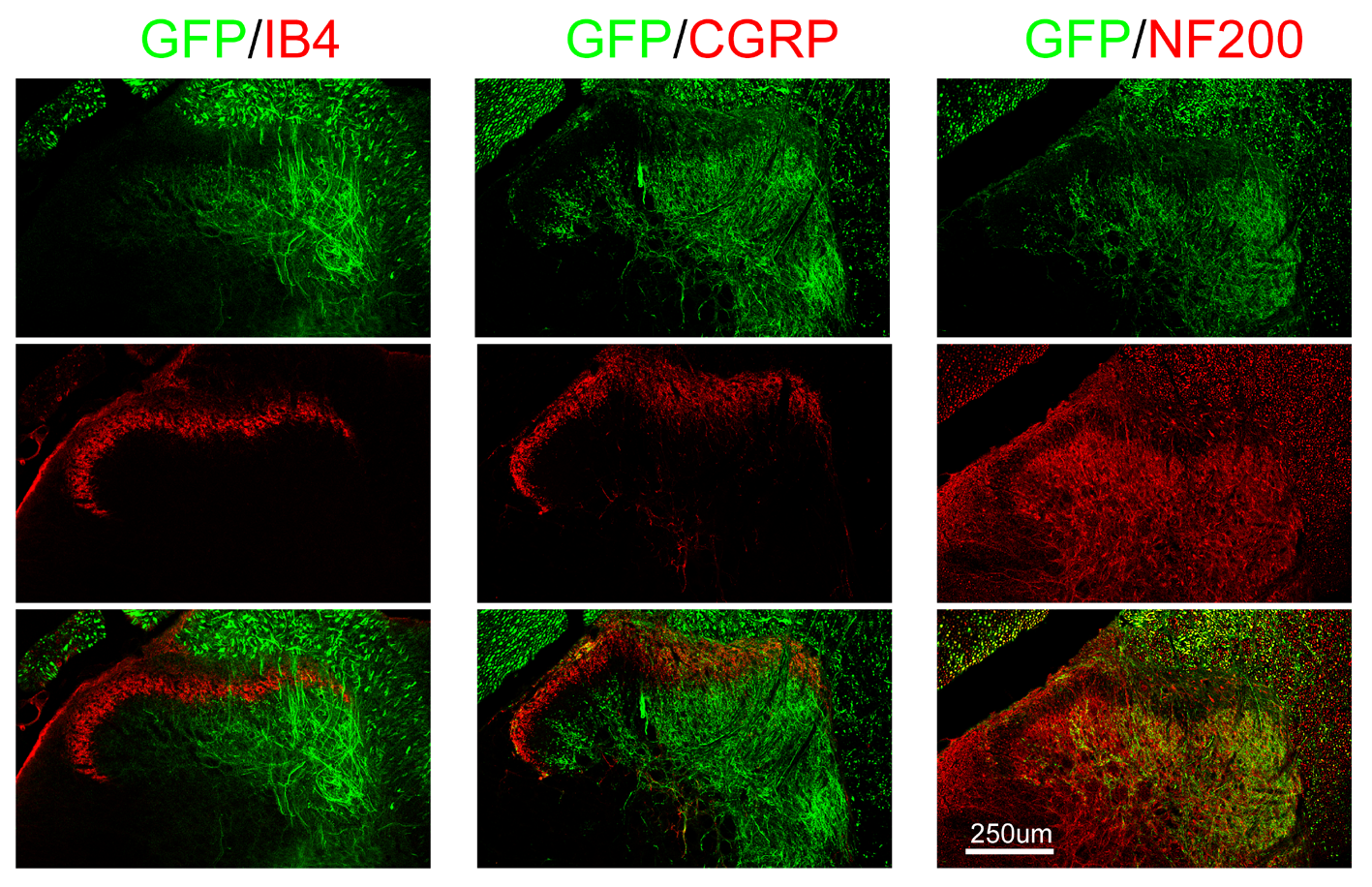

Supplement: Figure S1 — Co-labeling of GFP (green) with IB4, CGRP or NF200 (red) in the lumbar spinal cord at 2 week-post intrathecal AAV5 vector injection. Scale bar, 250 µm. (TIF) [file pone.0032581.s001.tif]

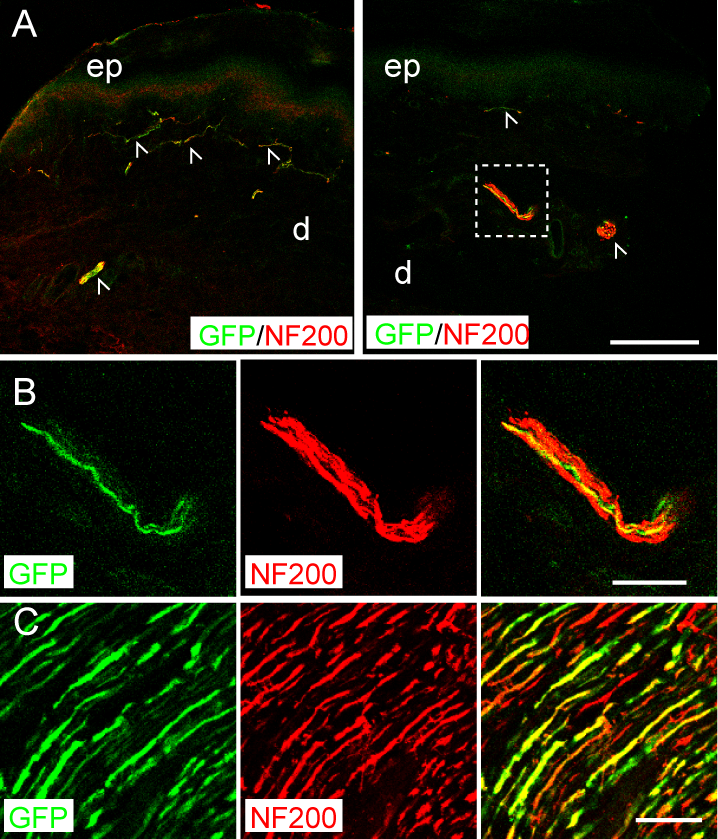

Supplement: Figure S2 — Expression of GFP (green) and NF200 (red) in (A and B) the glabrous skin of the hind paw and (C) sciatic nerves. ep, epidermis; d, dermis. scale bars, a, 200 µm; B and C, 50 µm. (TIF) [file pone.0032581.s002.tif]

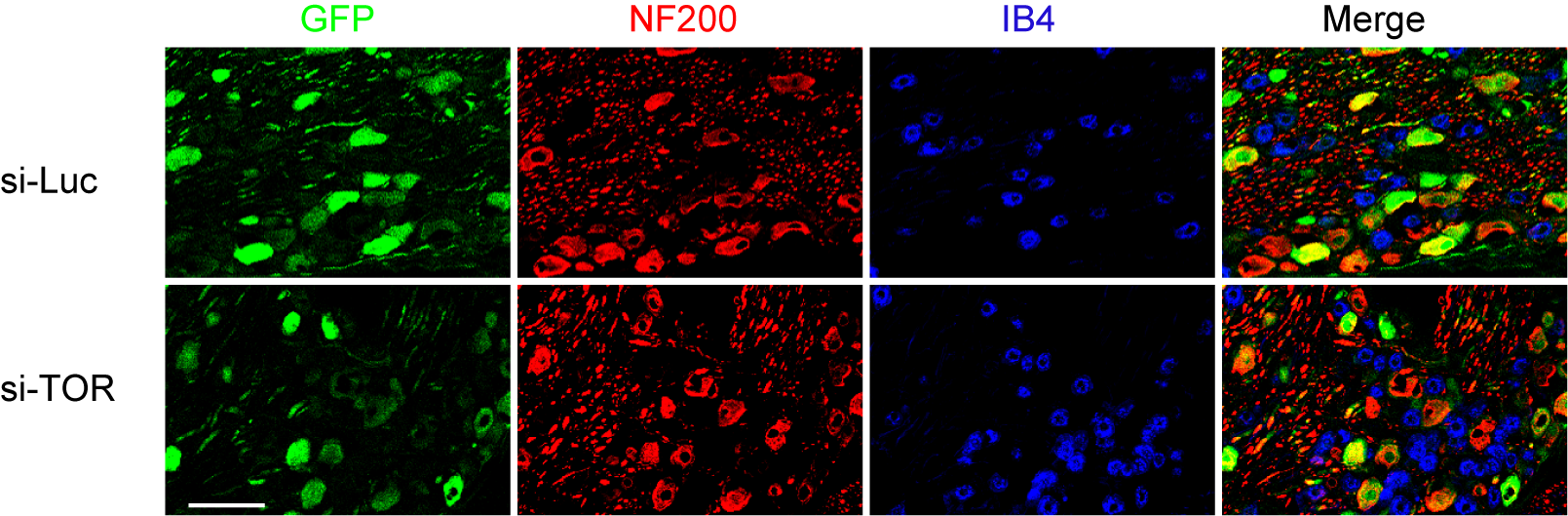

Supplement: Figure S3 — GFP (green) in lumbar DRG co-labeled with NF200 (red) and IB4 (blue) at two week following si-Luc or si-TOR vector administration. Scale bar, 100 µm. (TIF) [file pone.0032581.s003.tif]

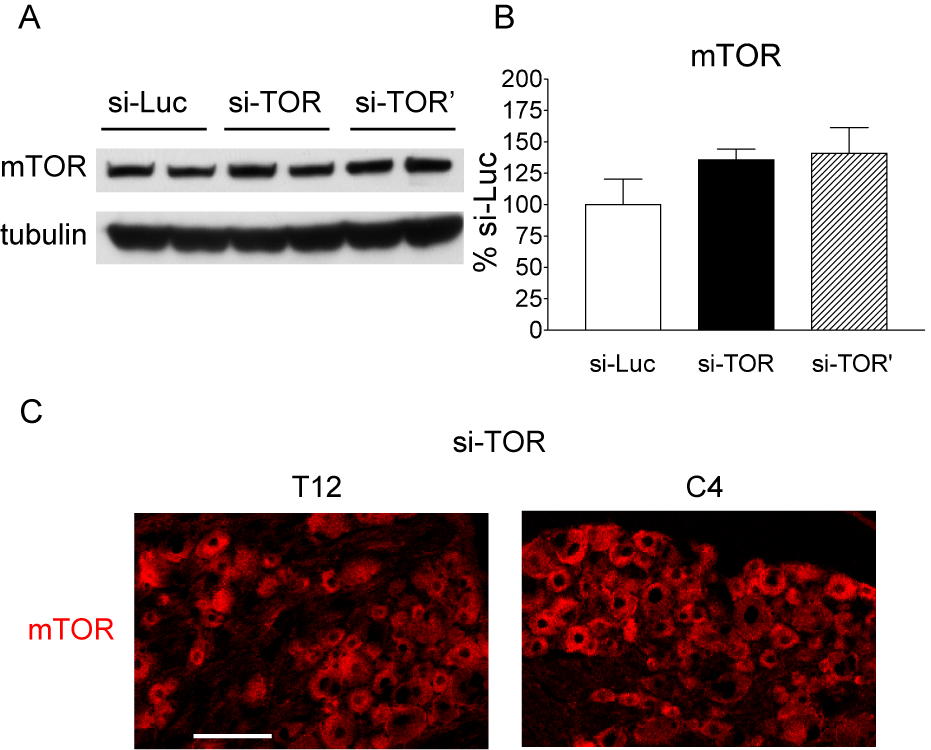

Supplement: Figure S4 — Expression of mTOR in the lumbar spinal cord and cervical and thoracic DRGs. A) Representative Western blots showing levels of mTOR and beta-tubulin in the lumbar spinal cord dorsal horn at 5 week following vector administration. B) Histogram represents the mean mTOR levels in respect to the control group (si-Luc). C) Confocal images of mTOR in T12 and C4 DRG 2 weeks following the si-TOR vector injection. Scale bar, 100 µm. (TIF) [file pone.0032581.s004.tif]
